# Scaling community-based services in Gauteng, South Africa: A comparison of three workforce-planning scenarios

**DOI:** 10.4102/phcfm.v10i1.1748

**Published:** 2018-05-31

**Authors:** Rod Bennett, Tessa S. Marcus, Geoff Abbott, Jannie F. Hugo

**Affiliations:** 1 Department of Family Medicine, University of Pretoria, South Africa

## Abstract

**Background:**

The introduction of community-based services through community health workers is an opportunity to redefine the approach and practice of primary health care. Based on best-practice community oriented primary care (COPC), a COPC planning toolkit has been developed to model the creation of a community-based tier in an integrated district health system.

**Aim:**

The article describes the methodologies and assumptions used to determine workforce numbers and service costs for three scenarios and applies them to the poorest 60% of the population in Gauteng, South Africa.

**Setting:**

The study derives from a Gauteng Department of Health, Family Medicine (University of Pretoria) partnership to support information and communication technology (ICT)-enabled COPC through community-based health teams (termed as ward-based outreach teams).

**Methods:**

The modelling uses national census age, gender and income data at small area level, provincial facility and national burden of disease data. Service calculations take into account multidimensional poverty, demand-adjusted burden of disease and available work time adjusted for conditions of employment and geography.

**Results:**

Assuming the use of ICT for each, a health workforce of 14 819, 17 925 and 7303 is required per scenario (current practice, national norms and full-time employed COPC), respectively. Total service costs for the respective scenarios range from R1.1 billion, through R947 million to R783 million.

**Conclusion:**

Modelling shows that delivering ICT-enabled COPC with full-time employees is the optimal scenario. It requires the smallest workforce, is the most economical, even when individual community health worker costs of employment are twice those of current practice, and is systemically the most effective.

## Introduction

There is renewed and significant growth in global interest in extending primary health care (PHC) services to communities through community health workers (CHWs).^1^ The present focus on CHWs is driven by the need to meet health service demands generated by the high burden of disease as well as by national and international public expectations of universal access to quality health care. It is also driven by the inability of facility-based, specialist and specialising health services to achieve the Millennium Development Goals (MDG), let alone the more ambitious health targets set out in Sustainable Development Goal 3 (SDG 3).^2^

Now, as in the 1980s, governmental and agency focus on CHWs tends to treat community-based services as a low cost add-on to existing health care systems rather than an opportunity to redefine the approach and practice of PHC.^2^ As a consequence, both internationally and in South Africa, CHW programmes are characterised by an often tangential relationship to the PHC system; limited planning and general under-resourcing; discretionary, short-term and arbitrary financing; little or restricted, short-course training; low or limited management, supervision and support; and poor and divisive terms and conditions of employment that are outside international and national norms.^3,4,5,6^ There is a concern that the renewed focus and effort around CHWs will inevitably fall short of possibilities and expectations if the above-recognised critical programmatic factors are not addressed.^2^

Community oriented primary care (COPC) is an internationally recognised approach to PHC. It combines the tenets of clinical care and public health into an integrated practice developed to meet the health care needs of poor, underserved and excluded people. COPC’s roots lie in the work of John B Grant and the Rockefeller Foundation in China, India and Java in the 1920s and 1930s. Community oriented primary care was first elaborated as an approach and a practice in Pholela, South Africa, by Drs Sidney and Emily Kark together with Edward and Amelia Jali. For 17 years, they and a growing team of health care professionals and workers were able to develop, sustain and expand its application in South Africa until they and the approach were crushed by the apartheid regime.^7^ Thereafter, it spread to countries and regions around the world where it remains an appropriate 21st century system response to the health challenges that continue to afflict millions of individuals, families, communities and societies.

In South Africa, COPC is part of an ongoing programme of transformation of health services^4,8,9^ defined by the government’s primary care reengineering framework, which includes the introduction of National Health Insurance (NHI).^10^ There, it is articulated as ‘the approach designed to operationalise the continuum of health care to and from homes, facilities and across disciplines, services and sectors in an Integrated Primary Health Platform (IPHP)’.^11^

The principles that guide COPC draw from the best available understanding and practice of inclusive PHC, namely geographically based, comprehensive generalist care that combines practice with science delivered through services that are equitable and integrated around users.^12,13,14,15^

In its contemporary application in South Africa, COPC is realised in communities through the creation of geo-specific community-based health teams, termed ward-based outreach teams (WBOTs). Ward-based outreach teams are made up of a number of CHWs who are managed by a team leader (termed outreach team leader [OTL]). Each team is attached to a health care facility (clinic, community health centre and district hospital) and is supported by facility and district-based health care professionals. Each CHW is assigned a defined number of households around the geographic community where she or he lives.^16^

Teams and individual health workers are supported by a purposively developed and tested mobile and web platform that has been effectively used by over 10 000 rural, peri-urban and urban community and other health care workers in Gauteng and other South African provinces (De Vos J 2017, personal communication, November 20). The ICT platform, called AitaHealth^™^, is designed to help identify individual and household health care needs, support quality service delivery, assist human and material resource management, support capacity development and enable effective monitoring and evaluation. In addition, and as in the past, work integrated learning is explicitly built into COPC practice in order to create progressive and cumulative capability and to ensure the provision of quality care. This network of people, services and systems constitutes the facility-linked community-based tier of a COPC integrated district health system.

Although information and communication technology (ICT)-enabled COPC is increasingly practised in Gauteng, its parameters of execution are constrained by the primary care reengineering framework developed by the National Department of Health (NDoH).^17^ According to this framework, WBOTs are an add-on to clinics. They function to extend the reach of existing facilities, the notional delivery end point of PHC. Extraneous to service and located at the bottom of the health care hierarchy, WBOTs are built on a casualised, predominantly female, part-time CHW labour force that is indirectly employed and nominally paid. Moreover, they predominantly depend on 20th century technology and training assumptions.

The national framework uses a normative approach to determine both the size and composition of teams (one OTL, six CHWs, four home-based carers and one data capturer) as well as the ratio of households to CHWs (one team per 6000 population).^17,18,19^ These norms; however, do not appear to be based on any recognised methodology that accounts for the population and geography of urban and rural settings, socio-economic disparities, health care needs or effective team management. In brief, the framework has both conceptual and practical challenges.

One of the key challenges is the problem of how to determine the way in which community-based teams can be allocated to communities. To address this problem, we have created the COPC planning toolkit. It has two components. The first is a mapping application. It links the population data of each small area to the nearest health care facility and calculates the distance of this small area population from the facility. The second is a computer model. It calculates, (1) workload, using national best-practice guidelines, (2) staffing need, applying the workload indicators of staffing need (WISN) methodology developed by the World Health Organization (WHO) and (3) the cost of implementation. In addition, explicit account is taken of the local burden of disease as impacted by socio-economic status. Nationally available data as well as experience and data from ICT-enabled sites in Tshwane district (Gauteng province) are also used. Following this methodology, the number of CHWs and the number and size of teams required to address the health needs of people who live in defined geographical communities are calculated.

This article presents the results of applying the COPC toolkit methodology to data from the five Gauteng province districts. It describes the costs of a community-based service in a COPC integrated district health system and compares these results to those of the existing approach that adds WBOTs to PHC facilities, using the total population living in a PHC facility catchment area as the sole criterion to determine numbers.^19^ The calculations used here have been refined in district workshops with service and management personnel across Gauteng.

## Research methods and design

### Determining community health worker and team numbers

Determining the human resources needed to provide a community-based service requires an understanding of the community, both in terms of its size and location, the time available to deliver services, the service need and the time required to meet it both relative to demand and in respect of each consultation.

The starting point is to understand the shape, size and characteristics of the community. In the COPC toolkit community is defined geographically, using Statistics South Africa’s (StatsSA) small area layer (SAL) demarcations (StatsSA 2013, personal communication, April).

Every small area has a number of households and a population. These are characterised socio-demographically using specific StatsSA age, gender and household size census data. Statistics South Africa’s full national household income data set is then used to determine the relative wealth and poverty of households in each small area as well as to calculate thresholds for each of the income quintiles. These income thresholds to determine the number of households in a small area that fall in each of the income quintiles.

Together, the sum of small area population profiles defines the catchment population and its socio-demographic characteristics, namely age, gender, households and income profile. As supervision and support of CHWs is critical to sustainable high-quality care, and this analysis is for the planning and costing of outreach teams, we differentiated between CHWs who are distal or proximal to their support structure. Outreach team leaders based in facilities more than 2 km from the CHWs they manage require a planned transport system to provide regular and close supervision. Also, travel time limits the effective size of their teams. We therefore created demographic profiles for proximal and distal areas for each PHC facility. Within distal areas, we further distinguished communities by population density. Unlike distal densely populated communities, remote communities are small, with less than 200 people per square kilometre. In order to ensure a community-based service, CHWs who work in remote communities therefore are considered to be employed part time in proportion to the size of the population that they serve.

The StatsSA small area data set provides population data for 4539 small areas in Tshwane (4459 proximal, 22 distal and 58 remote), 4290 in Ekurhuleni (4253 proximal, 15 distal and 22 remote), 5899 in Johannesburg (5893 proximal, 0 distal and 6 remote), 1420 in West Rand (1367 proximal, 17 distal and 36 remote) and 1475 in Sedibeng (1410 proximal, 23 distal and 42 remote). The sum of the proximal, distal and remote catchment populations and their characteristics (age, gender, households and income profile) thus provide the proximal and distal small area catchment population profiles, respectively, for each PHC facility.

According to the NDoH, in the national norms scenario, 6999 teams are needed to deliver WBOT services across the country. This figure was arrived at by applying a simplistic ‘number of households’ population model to determine workload. The scenario assumes a uniform target population of 6000 per WBOT and a uniform household and citizen allocation per CHW of 250 households and 1000 people.

In contrast, using the COPC toolkit, it is possible to determine team numbers by workload, taking into account both available working time and the time needed to deliver the service. Thus, the available working time for each CHW in the full-time employed COPC scenario was assessed using the WHO Workload Indicators of Staffing Need (WISN) methodology.^20^ Tshwane COPC empirical data were used to estimate the duration of each consultation. The anticipated number of consultations that each CHW would be required to make was estimated using the PHC reengineering guidelines for key health conditions, and was further refined by adjusting for the relative burden of disease. Thus, in this scenario, the multidimensional poverty index (MPI) was calculated for each sub-district and adjusted proportionally in relation to the percentage of quintile 1 households in the catchment populations of each facility.^21^ Also, the numbers of households in each small area were stratified (under 50, 50–100 and so on to over 600 households) in order to ensure equity, both in respect of service delivery needs and health care provider workload. Available working time and additional human and material resources were adjusted to compare ICT-enabled and traditional paper-based information systems.

### Determining the cost of delivering community-based services

Costing is based on the direct incremental costs of community-based service delivery in a COPC integrated district health system. It takes into consideration key factors that determine its chances of success, particularly remuneration, overhead costs, information collection and management, equipment, education and training, transport and infrastructure.

In terms of remuneration, pay scales for formally employed personnel are drawn from the 2017 Public Sector Commission Bargaining Chamber pay awards.^22^ Remuneration for contracted workers is based on the proposed national minimum wage (ZAR 20/h).

In terms of overhead costs and working arrangements, the following assumptions are made for all scenarios. Community health workers work in teams managed by team leaders (OTLs) to deliver community-based health services. Facility unit and sub-district contracting unit managers manage OTLs and oversee teams. Outreach team leaders and clinical supervisors are housed in existing infrastructure (e.g. clinics, CHCs or other facilities) without incurring overhead costs. Community health workers are based in and preferably drawn from their respective small area communities. Community health workers have at least two face-to-face contacts a week with the team leader and team leaders have at least two face-to-face contacts with a clinician (such as a professional nurse, a clinical associate, etc.). In addition, three facility-based CHWs provide patient point of care counselling and linkage to care services as part of the facility team.

In scenarios where community-based services use an ICT-enabled platform, team leaders and CHWs each have a dedicated device (phone or tablet) linked to the web. The platform provides bidirectional communication to support quality service and learning, human resource management, monitoring and evaluation, data integrity and system reliability. Each facility has a computer, printer and Internet access. In alternative scenarios where data and CHWs are managed through a paper-based system, available working time is reduced by an hour and visit time is increased by 5 min to allow for the slower pace of data capture, as well as file management and clocking on and off at the facility.

In terms of education, all teams are supported through ongoing work integrated learning to develop capacity and ensure quality of care. After initial training, each OTL and CHW receives 16 days on-the-job training each year. Current practice uses facility-based training, the costs of which include refreshment, transport and materials.

All teams and individuals are provided with the necessary equipment and materials to support service delivery and learning. Equipment costs are annualised and include maintenance and replacement. The cost of kits is based on a recent analysis by Gauteng province (Thomas, L 2017, personal communication, March 29). For other equipment, uniforms, leaflets, office supplies and so on, we used 2017 prices sourced from the Internet. Transport costs cover OTL distal and remote supervision and clinician or supervisory support.

The costing is based on the annual full operational cost of the teams.

## Results

For the purposes of this article, the COPC planning toolkit is applied to three scenarios, (A) current practice, (B) ICT-enabled COPC with full-time employment and (C) national norms, factoring in paper-based and ICT-enabled data and performance management systems accordingly. In all three scenarios, we assume the same population coverage and that CHWs perform similar tasks, with similar degrees of efficiency. Though we adjust working time to allow for current employment practices, we do not take into consideration known equipment and resourcing challenges or differences in work practices, productivity, down time, training levels, supervision, staff turnover or skills development. The relevance of these issues is partly covered in the discussion, although their impact on cost-effectiveness will be shown more fully elsewhere.

### Scenario A: Current practice

Current practice in Gauteng is a mixed model that combines facility-based WBOTs and the ICT-enabled COPC model developed in Tshwane that is being phased in across the province by the Gauteng Department of Health (GDoH) (Table 1). Community oriented primary care teams use AitaHealth^™^ on devices (phones, tablets or laptops). The remaining majority of WBOTs use a paper-based system. Data capturers input information gathered by them into the electronic system. Community health workers work in teams of 8-–15 workers. Enrolled nurses who lead and manage teams (OTLs) are based in facilities. They work full time for the GDoH. Outreach team leaders for WBOTs are expected to perform both facility and WBOT functions, while OTLs for COPC teams are expected to spend more time working with their teams. Community health workers are contracted for 200 days a year at ZAR 3500 per month. They are employed to work 6 hours a day in a 5-day week. Effective CHW work time; however, is reduced by 2 hours a day to accommodate facility-based in-service training and current conditions of employment that require them to sign in and sign out at base clinics at the start and end of each working day.

**TABLE 1:** A three-scenario comparison of coverage, resources, performance and costs of community-based services in Gauteng province.

Gauteng province	Current practice ICT: Scenario A	Current practice paper: Scenario A – paper based	Full-time employment ICT-enabled: Scenario B	National norms paper: Scenario C
**Coverage**				
**Total population**	**13 716 027**	**13 716 027**	**13 716 027**	**13 716 027**
Prioritised population	9 370 719	9 370 719	9 370 719	9 370 719
Facilities included	381	381	381	381
**Resources**				
Number of CHWs	10 877	15 706	6590	9656
Number of HBCs	2636	3823	-	6413
Number of OTL and/or teams	1306	1802	713	1856
**Total community service staff**	**14 819**	**21 331**	**7303**	**17 925**
Number of CHW facility linkage to care	1390	1886	797	1886
**Performance**				
**Total consultations**	**26 385 260**	**26 385 260**	**26 385 260**	**not known**
Consultations per capita	2.82	2.82	2.82	not known
Population per CHW	871	600	1815	984
Households per CHW	221	153	461	250
**Costs**				
**Total cost**	**947 665 941**	**1 532 026 445**	**783 486 376**	**1 349 154 557**
CHW cost	453 198 060	657 316 091	542 004 840	402 004 664
HBC cost	110 693 940	160 549 909	-	269 343 125
OTL cost	209 018 774	288 344 791	114 141 146	296 952 576
Linkage to care cost	58 396 800	79 220 400	66 799 793	79 220 400
Cost per CHW	87 825	97 891	150 673	140 955
Cost per capita	102	165	84	145
Cost per visit (consultation)	36	58	30	not known

CHW, community health worker; OTL, outreach team leader; HBC, home-based carer.

For this analysis, the rollout of ICT-enabled COPC teams across the province is assumed, while retaining the current employment arrangements. Given workforce instability as well as limited supervision and on-the-job training, each consultation is estimated to take 25 minutes, with travel time between proximal and distal consultations estimated at 5 and 10 min, respectively. Community health workers use a cell phone reporting system and have a full management, support and supervision structure. Through the first phase of scaling, they are allocated to households in poorer areas, where income quintiles 1–3 predominate. Every household is visited annually for health promotion and assessment. Service, follow-up and campaign consultations are based on identified need or condition guidelines. Home-based care is assumed to be carried out by not-for-profit civic, community or faith-based organisations.

Under Scenario A, Gauteng requires a community-based health workforce of 14 819. It is made up of 10 877 CHWs plus 2636 home-based carers (HBCs) organised into 1306 teams. Each CHW can be assigned around 221 (proximal), 202 (distal) or 83 (remote) households.

The total cost of an ICT-enabled COPC service to support all community-based health workers organised this way is ZAR 948 million, of which 57% is CHW and HBC costs and 88% overall human resource costs. The cost increases by 56% to ZAR 1532 million using a paper-based system. If, as in the NDoH model, HBC is deemed a separate task performed by a different workforce, the HBC cost is estimated at ZAR 111 million and 11.6% of the overall ICT-enabled service cost.

### Scenario B: Full-time employment, ICT-enabled community oriented primary care

In this scenario, CHWs are a stable component of the health care workforce, supported by continuous work integrated learning and closely performance managed (Table 1). Community health workers are employed by government according to national labour standards. They earn entry-level Department of Public Service and Administration (DPSA)-determined wages, adjusted for inflation (ZAR 6981 per month 2017/2018). They work 222 days a year, inclusive of 16 on-the-job training days carried out by OTLs and other health care practitioners. Their conditions of employment allow for a reduction in both time allocated to consultations (20 rather than 25 min) and turnover intervals between consultations (2–5 rather than 5–10 minutes).

They work with their assigned households in the community for 7 hs a day. They are allocated to households in quintiles 1–3 predominant communities. Home-based care services are carried out by CHWs. They sign in and sign out of work and are performance-managed through their devices.

In this scenario, Gauteng province requires a community-based health workforce of 7303. It is organised into 713 teams. Each CHW is assigned and services between 461 (proximal), 320 (distal) and 83 (remote) households providing comprehensive care, including home-based services, as appropriate.

The total cost of Scenario B is ZAR 783 million, of which 68% is CHW costs and 92% is overall HR costs.

### Scenario C: National Department of Health population norms

In this scenario, the team comprises six CHWs, four HBCs and a data capturer managed by a team leader using a paper-based system with facility computers (Table 1). Training, supervision and oversight are costed in the same way as the workload-based scenarios described above. We use current practice assumptions regarding visit and turnover interval time. Each CHW is responsible for 1000 people (equivalent to 250 households in quintiles 1–3).

According to this scenario, Gauteng requires a community-based health workforce of 17 925 people. Of the 16 069 care workers, 9656 are CHWs and 6413 are HBCs. They are organised into 1856 teams, each supported by one team leader and one data capturer. Each CHW is assigned and services between 250 (proximal or distal) and 86 (remote) households.

The total cost of applying the NDoH population norm scenario is ZAR 1349 million. Of this, 29% is CHW costs, 21% is HBC costs and 94% overall HR costs. If an ICT-enabled system is used, the total cost decreases by 17% to ZAR 1118 million.

In summary, both in terms of human resource requirements and costs, ICT-enabled COPC with CHWs employed full time (Scenario B) requires the least number of CHWs and community-based health teams and is the least costly.

## Discussion

Going to scale in a COPC integrated district health system is premised on starting from a base that addresses the factors that have been consistently described as being critical to the successful extension of primary health services through community-based services. To this end, we have assumed that personnel and functioning preconditions, including the recruitment, employment, training, supervision, infrastructure and resourcing requirements, as well as community mobilisation apply to all scenarios.

Modelling shows that the NDoH population norms scenario requires a very large workforce (17 925). It is the most expensive of the three scenarios, costing 42% more than current practice (Scenario A) and 72% more than the full-time employment ICT-enabled COPC (Scenario B). The results also show that the calculated workload would only allow each CHW to cover between 83 and 153 households, whereas the national norms model dictates they cover 250. The implication of this is that they could only achieve a maximum of 61% coverage, and this at greater cost. If a paper-based system is used, Scenario A (current practice) would increase the required personnel from 17 925 to 21 331 to provide the coverage.

In terms of workforce numbers, current practice (14 819) and the population norms scenarios (17 925) perform comparatively, as both are built on the same casualised, low-pay, part-time and poor conditions of employment labour practices. The workforce size and costs of the former are somewhat less expensive, primarily because ICT is used to increase efficiency.

The full-time employment, ICT-enabled COPC scenario (B) requires the smallest workforce (7303), approximately 49% and 41% of the other two scenarios. It is also the least costly of the three scenarios. Because CHWs work full time, with ICT-supported human resource management, the number of households they can be expected to serve significantly increases. Workforce numbers are also kept down by adopting a generalist approach to service delivery that includes HBC support.

The comparison does not take into account the known risks and inefficiencies of Scenarios A (current practice) and C (NDoH norms). In Gauteng and elsewhere, intermittent and persistent disputes over pay and conditions of employment have led to unrest, attrition and general workforce instability, inconsistent and lengthy interruptions in service delivery, poor quality assurance and costly litigation. Scenario B (full-time employment) is likely to significantly mitigate these known risks. However, the extent to which it can reduce system instability, build CHW and team capacity to provide quality care and improve the value and credibility of the service has yet to be determined.

Methodologically, the COPC toolkit brings together well-used and widely respected international approaches to human resource planning in health care. It also draws on national best-practice guidelines in respect of conditions as well as national employment policies. Furthermore, the toolkit parameters are calibrated from actual practice, using the most up-to-date data available as well as experience from the field. By applying real data and using tested and well-established methodologies, the COPC toolkit is able to generate reliable outcomes and is relatively robust. This said it can be expected to be further refined through ongoing application in the field.

## Conclusion

The potential to change health and care in a district health system is significantly increased by the combination of ‘high-touch’ COPC supported by 21st-century, ‘high tech’ biomedical and information and communication technologies. Modelling the assumptions about the nature, size and costs of creating community-based primary care services is essential to make rational and informed policy decisions. The COPC planning toolkit can be used to assist the planning and mobilisation of resources to go to scale in an equitable and sustainable way as a managed process over time. Although illustrated here through a South African provincial example, it can be applied to larger or smaller contexts where there are local data, a health care system and a framework for health care service delivery.

Modelling the community-based tier in a COPC integrated district health system that extends to and from the home in South Africa shows that the ICT-enabled, full-time employment scenario. It requires the smallest workforce and is the most economical. This is the case even though the full cost of employment of each CHW is twice that of the currently contracted CHW. It shows that it is possible to achieve the same levels of activity and coverage, including HBC, with a smaller, regularised workforce. This saving in personnel and costs is achieved through productivity gains that come with workforce stability, full-time employment and ICT-enabled human resource development and performance management. In addition, the application of the optimal scenario can be expected to generate improvements in individual, household and community health and health literacy as well as system access, effectiveness and efficiencies that accompany continuity of personnel, services and information.
